# Diseases of the Stomach

**Published:** 1910-03

**Authors:** 


**Diseases of the Stomach.** By S. H. HABERSHON, M.A., M.D. Pp. xii, 565. London: Cassell & Company, Limited. 1909.

This book will be found a very useful and trustworthy guide to diseases of the stomach, and we can recommend it to our readers. We think that the author has been successful in his intention of supplying a concise and intelligible description of these diseases. The book is evidently the product of long-continued work and thought, and therefore is free from the marks of hasty writing which disfigure many modern medical treatises. On page 55 the introduction of small calories is confusing to the reader. There is an excellent account of the anatomy and physiology of the stomach, and the two chapters on diet in health and disease will be found very useful. The author has devoted two chapters to the general symptoms of stomach diseases and their mode of production, which make for clearness, although they involve some little repetition when he comes to deal with special diseases. The account of modern methods of investigation and of the ways in which they should be carried out is well written; the directions are clear and can be easily followed. On matters of treatment the author's advice generally is sound and well considered, but in the treatment of gastric ulcer we cannot agree with his recommendation of rectal feeding for two weeks after severe hemorrhage. This starvation plan has been superseded by better methods. The section on gastric cancer is especially valuable. We would suggest that in future editions, which we hope will be called for, the subject of subdiaphragmatic abscess should be dealt with at greater length. There are numerous illustrations and many coloured plates; those illustrating the anatomical relations of the stomach and cancer of the organ call for special commendation. The book is of a convenient size and form and the print clear.

